# Differences of Curing Effects between a Human and Veterinary Bone Cement

**DOI:** 10.3390/ma12030470

**Published:** 2019-02-03

**Authors:** K. M. Z. Kallol, M. Motalab, M. S. Parvej, P. R. Konari, H. Barghouthi, M. Khandaker

**Affiliations:** 1 Department of Mechanical Engineering, Bangladesh University of Engineering and Technology, Dhaka 1000, Bangladesh; rahilkallol@gmail.com (K.M.Z.K.); mtipuz@yahoo.com (M.M.); kowshikparvej@yahoo.com (M.S.P.); 2 Department of Engineering and Physics, University of Central Oklahoma, Edmond, OK 73034, USA; pkonari@uco.edu (P.R.K.) hbarghouthi@uco.edu (H.B.)

**Keywords:** titanium, PMMA cement, mechanical properties, exothermic temperature, orthopedics

## Abstract

The goal of the study is to understand how the curing characteristics of a human bone cement (HBC) and veterinary bone cement (VBC) influence the mechanical behavior of each cement and cement bonding with an implant. This study hypothesizes that the curing temperature and time influence the mechanical properties of the cement adjacent to the implant, which resulted in the variability in bonding strength between the implant and cement. To test this hypothesis, this study measured the exothermic temperature, flexural strength, hardness, and morphology of a HBC and VBC at different curing times. In addition, this study measured shear strength at the interfaces of implant/HBC and implant/VBC samples during static and stepwise cyclic tests at different curing times. This study used Stryker Simplex P and BioMedtrix 3 poly methyl methacrylate (PMMA) as an HBC and VBC, respectively. This study cured HBC and VBC cement for 30 and 60 min and then conducted flexural, hardness, and interface fracture tests to evaluate the curing effect on mechanical behavior of each of the cements. This study found that the curing time significantly increases the values of flexure and hardness properties of each cement and shear strength of implant/HBC and implant/VBC (*p* < 0.05). This study observed a difference of curing time and temperature between HBC and VBC. This study also observed a significant difference of surface porosity at the interface of implant/HBC and implant/VBC interfaces. The variability of mechanical properties between HBC and VBC due to the differences of curing conditions may influence the bonding of cement with the implant.

## 1. Introduction

PMMA bone cements commonly used in human and animal orthopedic surgeries. The current most commercially available human PMMA bone cements are Cobalt (Biomet, Inc., Warsaw, IN, USA) [1], Simplex (Stryker, Inc., Kalamazoo, MI, USA) [2], and Palacos (Heraeus Company, Hanau, Germany) [3]. The current most commercially available animal PMMA bone cements are BioMedtrix [4] and Jorgensen Labs veterinary bone cement [5]. One of the major drawbacks of using PMMA cements for those surgeries is strong exothermic temperature that happens during the curing of PMMA cement [6]. Thermal stresses resulting from the shrinkage of polymethyl methacrylate (PMMA) bone cement have been implicated in the formation of cracks in cement mantles following total hip arthroplasty [7]. High stress intensity is inherent at the corner of the bi-material joints due to both thermal and mechanical loading [8]. According to Reedy [9], stress intensity can exist at an interface corner within the context of both elasticity and work hardening plasticity theory. Therefore, the initiation and propagation of cracks from the bi-material interface is a major problem in the design of bi-material joints [10]. The strength of a bi-material specimen depends on the material and geometric properties of the joining materials [11]. The thermal stresses, which are caused by the exothermic temperature difference, can influence the material properties of cement and fracture energies at the implant-cement interface [12]. The magnitude of the developed stresses during the curing can be very large and may have a significant influence on the bonding of the interface, which is unknown as of yet. The interfacial mechanics at the implant/cement interface is a critical issue for implant fixation and the filling of bone defects was created by tumors and/or their excision [13]. The fixation of human and animal bone cement with implants may not be the same, since the mechanism of curing of the cement is different. This study will investigate how, for a HBC and VBC with different curing conditions, the yield differentiates the surface and mechanical properties of the titanium/cement at static and cyclic loading conditions. This study was conducted based on three research questions: (1) Is there any significant difference in the morphological behavior occurs due to the difference of curing time of a bone cement? (2) Is there a significant difference in the mechanical properties of bone cement occurs due to a difference of curing conditions? (3) Is there a significant difference in the bonding of an implant with bone cement that occurs due to a difference of curing conditions during static and cyclic loading?

## 2. Materials and Methods

### 2.1. Materials

This study used Stryker Simplex^®^ P bone cement (Kalamazoo, MI, USA) as a HBC and BioMedtrix 3 veterinary bone cement (Whippany, NJ, USA) as a VBC and titanium (Ti) alloy (Ti-6Al-4V ELI, ASTM B 348 standard, grade 23, biocompatible) of dimension 76 mm long × 3.96 mm diameter as an implant. This study purchased the Ti alloy from Supra Alloys (Camarillo, CA, USA). Among the various Ti alloys, this study used Ti-6Al-4V Eli because of its better physical and mechanical properties in comparison to pure Ti for orthopedic surgeries.

### 2.2. Experimental Design

This study conducted exothermic temperature, flexural, and hardness tests on HBC and VBC samples at the two different curing times. The study conducted pullout static and stepwise cyclic tests on Ti/HBC and Ti/VBC samples. The pre-load resulted during the curing of Ti/HBC and Ti/VBC samples was measured during the pullout static tests. This study also determined the roughness of cement joining the implant after the pullout static tests to measure the morphology of the cement near the interface between the implant and the cement. This study cured each cement for 30 and 60 min before conducting the above tests. The reason for the selection of the above curing times was that, within 30 min, complete curing of both HBC and VBC cement occurs.

### 2.3. Sample Preparation and Experiment

#### 2.3.1. Exothermic Temperature and Curing Time

**Figure 1:**
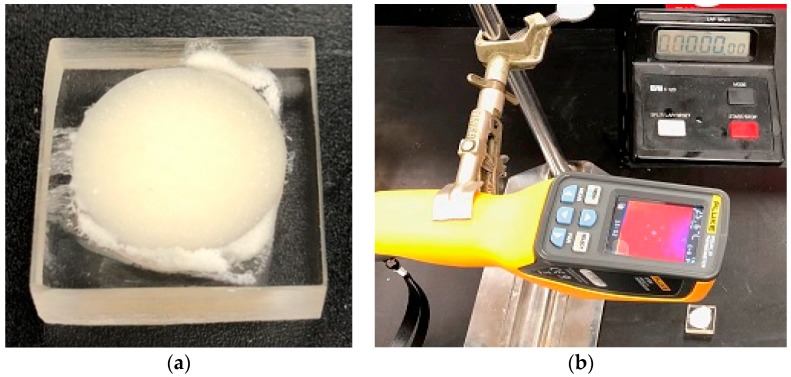
(**a**) Samples and (**b**) setup used for the measurement of exothermic temperature rise of cement.

An acrylic sheet having a hole of 1 inch in diameter and 1/4 inch in depth was used to cure each group of cement for measuring the exothermic temperature and curing time (Figure 1a). The same amount of powder and monomer was prepared for each group of samples to fill the hole on the acrylic. When the cement is semi-liquid phase, each cement poured on the well and was visualized by Fluke VT04 Visual Infrared Thermal sensor (Everett, WA, USA) (Figure 1b). The distance between cement and sensor (30 cm) was identical for all samples. Concurrently, a board pin poked the cement to measure the time required for complete curing of cement, which was determined when the board pin was unable to poke through the cement. The thermal sensor captured the exothermic temperature every 30 s until the drop of exothermic temperature. This study used a low-speed diamond saw machine (Buehler Isomet 11-1180-300, Lake Bluff, IL, USA) for longitudinal cross sectioning of the exothermic test samples. Hitachi 3000 scanning electron microscope (Tokyo, Japan) and Profilm 3D optical profiler (Filmetrics, San Diego, CA, USA) scanned the cut surface of the sectioned sample to examine internal morphology of cements.

#### 2.3.2. Three-Point Bend Test

**Figure 2:**
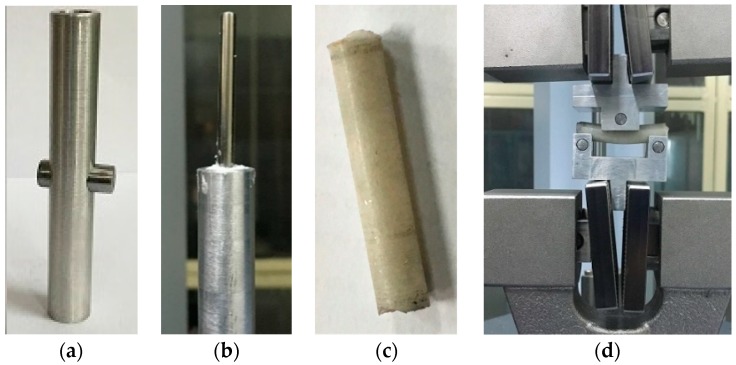
(**a**) Aluminum mold used to prepare Ti/cement samples for 3PB and pull-out tension tests. (**b**) A fabricated Ti/PMMA samples. (**c**) A 3PB cement samples prepared by removing the rod and aluminum rod and (**d**) a 3PB bend experiment on a prepared sample.

This study prepared a hollow cylindrical aluminum holder (length = 80 mm, outside diameter = 8.4 mm, and inside diameter = 8 mm) as shown in Figure 2a for the three-point bend tests. A rod pushed into the side hole of the holder so that cement can be cured in the hole of the cylinder without leakage. The top gripper of Shimadzu ASG -X series universal testing machine (UTM) (Shimadzu, Kyoto, Japan) fastened with a mirror polished Ti implant, whereas the bottom gripper of UTM was fastened with the aluminum holder. The inside surface of the aluminum holder was polished and lubricated with SAE 30 grade oil so that a low amount of push force is required for breaking the interface between cement and aluminum after curing. The top gripper of the UTM that contains the implant was slowly lowered so that the implant touches the top of the side rod. Each group of cement was prepared by mixing 0.62 g of PMMA powder and 310 mL of MMA monomer. The cement was hand-mixed and poured in the gap between the implant and holder. Figure 2b shows a cured PMMA with Ti in the holder. After the curing of HBC and VBC for a specific amount of time, the Ti rod were pulled off from the cement by moving up the Ti rod with a rate 1 mm/min. To remove the cylindrical cement sample from the aluminum holder, a 7.5 mm diameter rod was mounted at the top gripper of UTM that pushed off the top edge of the cement. Figure 2c shows a cylindrical PMMA sample that was used for a 3PB test. Three-point bend tests were performed on the cured samples using a custom made supporter and indenter (Figure 2d). Two steel rollers were press-fitted in the supporter at a distance equal to the span length (32 mm). A steel roller was press-fitted in the indenter at the center. The specimens were mounted on the custom-made 3PB indenter and supporter in the test stage during the flexural tests. Shimadzu ASG-X series universal testing machine (UTM) (Shimadzu, Kyoto, Japan) was used for the flexural tests (Figure 2d). The load and displacement were continuously recorded using trapezium X software (Shimadzu, Kyoto, Japan) until the failure of the specimens.

Flexural strength, *σ_b_*, was calculated using Equation [14].

(1) $$\sigma_{b}=\frac{P_{max}SR_{0}}{\pi \left(R_{0}^{4}-R_{i}^{4}\right)}$$

where *P_max_* is the ultimate load (force at failure), *S* is the span length (32 mm), *R*_0_ outer radius of the cylindrical sample, and *R_i_* is the inner radius of the cylindrical sample.

#### 2.3.3. Hardness Test

**Figure 3:**
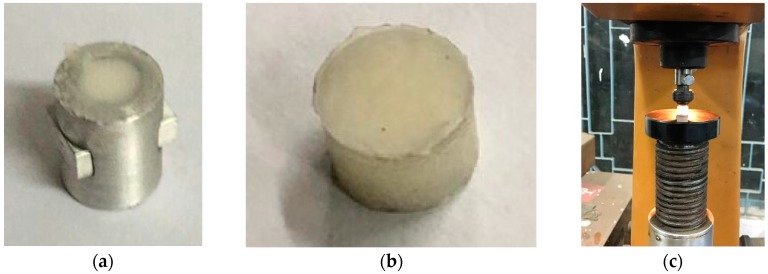
(**a**)Hardness test sample holder, (**b**) a fabricated sample that is tested to find the Rockwell hardness number, and (**c**) hardness test setup.

A hollow cylindrical aluminum holder (length = 30 mm, outside diameter = 8.4 mm, and inside diameter = 8 mm) was prepared for hardness test samples (Figure 3a). A 30 mm length and 8 mm diameter PMMA was produced following the same 3PB test samples preparation protocol, as described above. Figure 3b shows prepared PMMA samples for a hardness test. Indentations were done at three different places on each sample for the selected curing times. Rockwell hardness number (scale R) was read out from the scale from each indentation. According to this scale, a 1/2 steel ball was used, 30 kg of preload, and 60 kg of test force was applied. The load was applied for 15 s. The test was conducted at room temperature. The same test condition was applied for both HBC and VBC samples.

#### 2.3.4. Pull-Out Tension Test

This study used another custom-made cylindrical aluminum holder with the same dimension as the 3PB test holder. The inside surface of hole in the aluminum holder was threaded to create a higher amount of contact between cement and aluminum so that the breakage of the cement occurs at the implant/cement interface instead of cement/aluminum holder interface. Ti/cement samples for the pull-out tension test were prepared using the same method as 3PB test samples. After curing each group of cement with a specific time, the pullout static test was performed on the Ti/cement samples at a strain rate of 0.05 mm/s until the break of the Ti rod from cement. The maximum shear strength was calculated by dividing the force at the point of failure, *F*_max_ by the surface area of the implant in contact with the cement, 2π*R_i_L*, where *R*_*i*_ is the diameter of the implant and *L* is the length of implant in contact with the cement.

To understand the morphology of the cement along the interface of Ti and cement, surface roughness of each group of pullout tension samples were measured inside the curved surface of cement after the pull-out test. The Buehler Isomet low speed diamond saw machine (Buehler, Lake Bluff, IL, USA) cut the pullout tension sample transversely into a 30 mm long block. The saw machine further cut each block longitudinally in half. The inside curved surface of the sectioned sample was scanned by Profilm three dimensional (3D) optical profiler from Filmetrics, Inc. (San Diego, CA, USA) [15].

#### 2.3.5. Pull-Out Cyclic Test

The pullout cyclic test samples were prepared the same way as the pull-out tension test. The difference between the pull-out tension under static and cyclic loading was the type of loading applied to the Ti/cement samples. Stepwise cyclic pull out tension force was applied on Ti/cement samples using the same Shimadzu AGS-X series universal testing machine (UTM) (Shimadzu, Kyoto, Japan). Each group of samples after curing for 60 min, UTM recorded the residual load exerted from the cured cement to Ti. Each sample was pre-loaded with the following cyclic test parameter in the UTM: mean load = 20% of a residual load from curing, amplitude = 20% of the mean load and frequency = 2 HZ for 1000 cycles. After finishing pre-loading cycles, cyclic tests were repeated for another 1000 cycles with an increment of 50 N mean load using the mean load, *F_i_*_+1_ = *F_i_* + 50 N, amplitude = 20% of *F_i_*_+1_ and frequency = 2 HZ, where *i* = 0,1,2… The cyclic test cycles continued until the break of the specimen and the total number of cycles for failure of the Ti/cement interface was calculated.

### 2.4. Statistical Analysis

To determine whether there is a significant difference in the means of different experimental values between HBC and VBC groups, independent samples *t*-tests were performed based on unequal variances. All data were presented in the literature as mean ± standard error of the mean (SEM) (number of samples (*n*)).

## 3. Results

### 3.1. Exothermic Temperature

**Figure 4:**
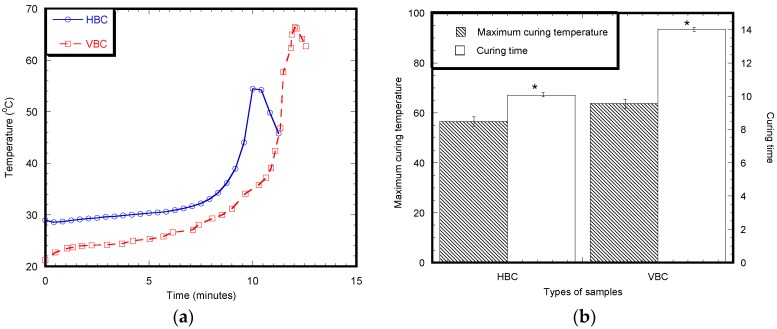
(**a**) A representative graph showing time versus temperature graph of an HBC and VBC specimen. (**b**) Curing characteristics (maximum curing temperature and time of HBC and VBC cements with respect to time). Data presented as means ± standard error of mean, *n* = 3 for each group of samples. Note: * *p* < 0.05 (compared to HBC).

Figure 4a shows the variation of curing temperature with respect to time for an HBC and VBC samples. All samples showed the similar characteristic of the temperature increase to a peak temperature, *T*_max_, and temperature decrease after *T*_max_. It is also evident from the graph that the types of cement influenced the time to reach *T*_max_. The time to reach *T*_max_ was lower for HBC samples compared to VBC samples. VBC showed higher *T*_max_ compared to HBC. Figure 4b reports the time required for the curing of HBC and VBC cement. The results show that it takes 8 min for a complete cure of HBC, whereas it takes 14 min for a complete cure of HBC.

**Figure 5:**
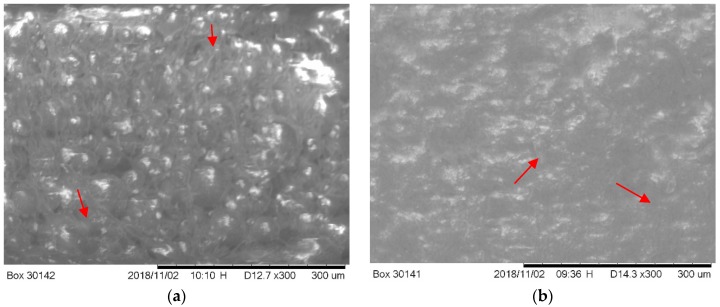
Scanning electron microscope images of a longitudinal sectioned HBC (**a**) and VBC (**b**) samples. Red arrow in the figure shows the presence of voids in the cement.

There is a difference of internal morphology between longitudinal sectioned HBC and VBC samples observed from the exothermic temperature test samples (Figure 5). SEM images show larger amounts of voids in HBC samples (Figure 5a) compared to VBC samples (Figure 5b). Although the study did not find any significant difference of *S*_a_ (HBC: 0.409 ± 0.009, *n* = 3 and VBC: 0.411 ± 0.026 *n* = 3) and *S_q_* (HBC: 0.582 ± 0.024, *n* = 3 and VBC: 0.534 ± 0.025 *n* = 3) values between HBC and VBC samples (*p* > 0.05).

### 3.2. Flexural Tests

**Figure 6:**
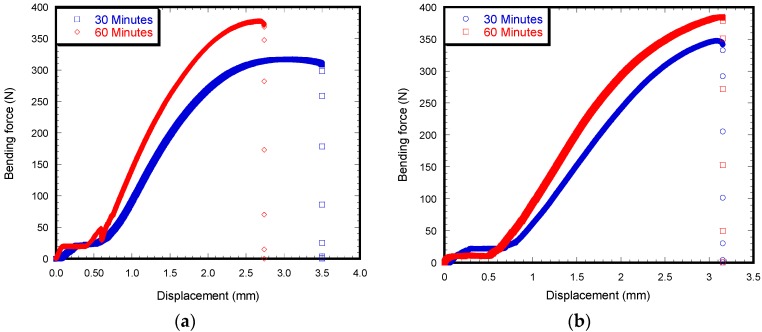
Load-displacement curve of a HBC (**a**) and VBC (**b**) samples during the 3PB tests.

Figure 6 shows the load-displacement behavior of an HBC and VBC sample for different curing times. The figure shows that, when curing time increases from 30 min to 60 min, the maximum bending load increase for both cements. However, the values of maximum bending load was higher for VBC samples compared to HBC samples. The c of load-displacement curves for HBC and VBC samples were different at different curing temperatures. The stiffness increases as the curing time is increased. However, ductility decreased when increasing the curing time. Therefore, the curing time has more impact on the bonding properties for HBC compared to VBC cement. Table 1 reports a comparison of the average maximum bending forces between 30 min and 60 min curing time for multiple numbers of tests. The results show that, as the curing time was changed from 30 to 60 min, the average bending strength increases by 24% for HBC and 7% for VBC. The difference of bending strength between HBC and VBC samples for both 30 and 60 min cured samples that are significant (*p* < 0.05).

### 3.3. Hardness Test

The study observed a change in hardness for both samples with the change of the curing time (Table 1). The Rockwell R hardness number increased by about 23.44% as the curing time increased from 30 min to 60 min for HBC, whereas the increase of Rockwell R hardness number was 69.05% for VBC. For both curing times, the values of hardness number of HBC samples was higher when compared to VBC samples.

### 3.4. Pull-Out Tension Test on Ti/Cement Samples

**Figure 7:**
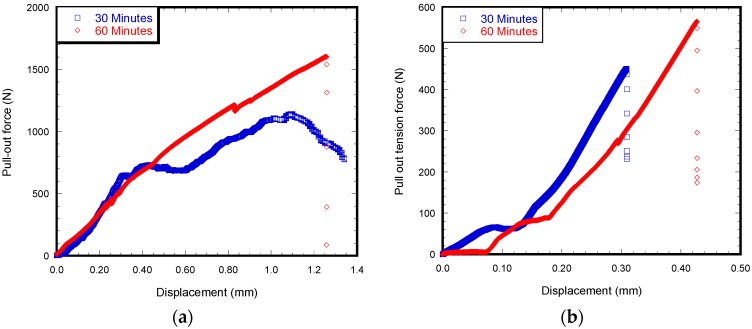
Load-displacement graph for an implant/cement during a pull out tension test under a static loading condition with respect to curing time made with HBC (**a**) and VBC (**b**).

Figure 7a,b show load vs. displacement curves of the Ti/HBC and Ti/VBC specimens at two different curing times from the pull out tension tests. Although both samples show load increases with displacement until fracture of the interface, there is a clear evidence that curing time and cement type influenced the fracture load. The study found that it takes 854 ± 44 N to break implant/cement interface, when the curing time is 30 min. As the curing time increased from 30 to 60 min, the force also increased to 1534 ± 36 N. The experiment on Ti/VBC found that it took 423 ± 20 N to fracture the interface, when the curing time was 30 min. As the curing time increased from 30 to 60 min, the force also increased to 526 ± 16 N.

Table 1 also summarizes the calculated results of the average pull out shear strengths for HBC and VBC at the two different curing times. It is clear from the data that, when the curing time is changed, the average pull-out shear strength increases significantly. There is a significant difference of the pullout shear strength after 60 min of curing between HBC and VBC samples (*p* < 0.05).

**Figure 8:**
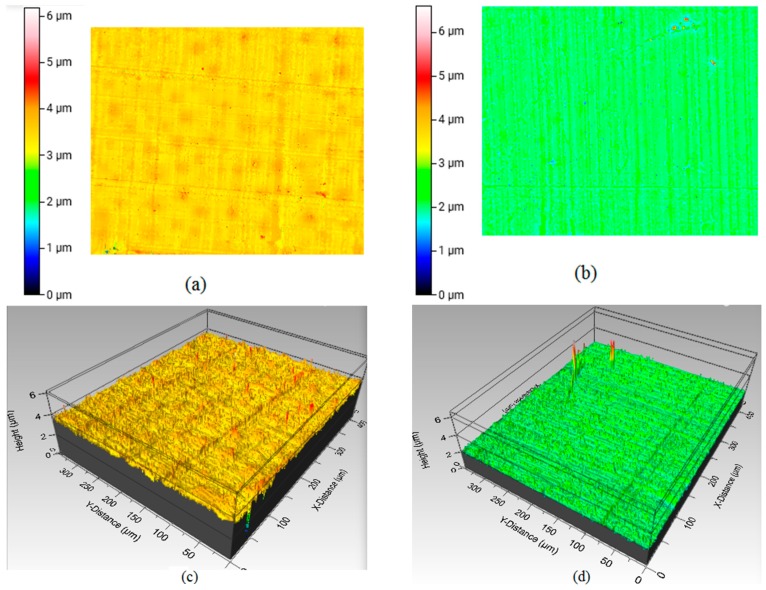
Two-dimensional views of the scanned surface profile: (**a**) HBC and (**b**) VBC sample. Three-dimensional views of scanned surface profile: (**c**) HBC and (**d**) VBC sample.

Figure 8a,b show the surface topographical view of an HBC and VBC samples from pull-out tension tests. The dimension of scanned images of the corresponding samples with a 3D surface profile is shown in Figure 8c,d. For both HBC and VBC, the differences of values of *S_a_* and *S_q_* for different curing temperatures were not significant (*p* > 0.05), but there was a significant difference of *S_a_* and *S_q_* values observed between HBC and VBC (Table 2).

### 3.5. Pull-Out Cyclic Test on Ti/Cement Samples

**Figure 9:**
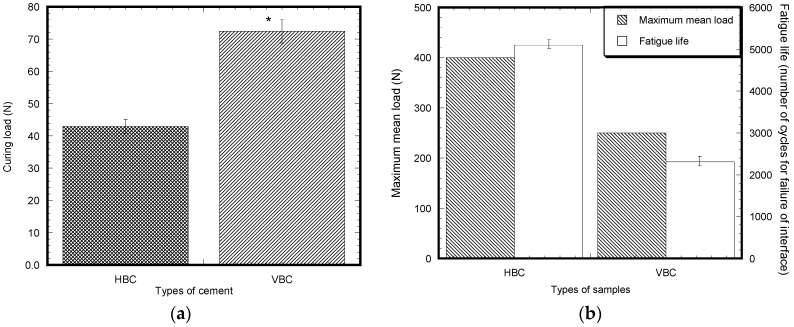
(**a**) Bar diagram of the variation of tension pre-load resulted after 60 min of curing Ti/HBC and Ti/VBC samples. Data presented as means ± standard error of mean, *n* = 5 for each group of samples. Note: * *p* < 0.05 (compared to HBC). (**b**) Bar diagram of the variation of pull cyclic test results of Ti/HBC and Ti/VBC samples due to the variation of the types of cement. Data presented as means ± standard error of mean, *n* = 3 for each group of samples. Note: * *p* < 0.05 (compared to HBC).

A significant difference of tension preload before the start of pull out cyclic tests on Ti/HBC and Ti/VBC were observed for every sample after 60 min of curing in the UTM (Figure 9a). It was typically 42.95 ± 2.70 N for HBC and 72.45 ± 8.91 N for VBC after 60 min of curing. This study found that 400 ± 80 N cyclic load and 5099 ± 52 number of cycles were required for the failure of Ti/HBC samples, whereas 200 ± 50 N cycle load and 2309 ± 43 number of cycles was required for the failure of Ti/VBC samples after 1 h of curing (Figure 9b). The values of the number of cycles of interface fracture was 2.2 times higher for Ti/HBC samples when compared to Ti/VBC samples.

## 4. Discussion

The study shows that VBC has inferior mechanical and curing properties when compared to HBC. Therefore, it requires further research to improve mechanical and curing properties of VBC. One of the potential ways to improve the properties is to use appropriate amounts of additives and alternative monomer with an HBC. Our earlier studies of adding chitosan, hydroxyapatite, and silica additives with an HBC shows a significant increase of mechanical and biological properties of the additive incorporated HBC compared to the HBC without additives [16,17].

When comparing the effect of cement type and curing time effect on bending strength and pull-out shear strength, it was found that the curing time has more impact on the pull-out shear strength than on the bending strength between HBC and VBC. The difference of pullout shear strength between Ti/HBC and Ti/VBC was 192% while the difference of bending strength between HBC and VBC was 6%. The observed difference of the flexural strength between HBC and VBC samples at different curing time may be due to the difference of internal morphology of cement during curing (Figure 6). We observed short and round voids in HBC samples, whereas long and elongated cracks were observed in VBC samples (Figure 5b). This study found a significant difference of surface morphology (*S_a_* and *S_q_* values) at the inside curved surface of cement samples between HBC and VBC (Figure 8 and Table 1). The observed difference of the shear strength between Ti/HBC and Ti/VBC samples at different curing times and loading conditions (static and cyclic), as depicted in Figure 7 and Figure 9, may be due to the difference of a residual pre-stress adjacent to the interface. When cement was curing, it applied pre-load to titanium, which was observed during the pullout cyclic tests from UTM (Figure 9a). The study observed the change in mechanical properties (ductility and hardness) of HBC and VBC with the change of the curing time, which might influence the bonding of the implant with the cement [12]. In fact, the study observed hardness in Rockwell R scale has increased by about 69% for VBC from 30 min to 60 min, where the increase was only 23.44% for HBC as the curing time increases from 30 min to 60 min. These results can be justified with the fact that cooling rate controls thermal residual stress that influence the bonding strength of implant/cement samples.

The novelty of this study is the comparative study to determine the relative influence of curing time on the mechanical properties of traditional HBC and VBC for orthopedic applications. This study also developed a novel experimental setup for all mechanical tests. The novelty of the setup is that the experiment can be conducted at a consistent condition. Additionally, the cured sample blocks for the setup can further be used for various mechanical testing. The results of the study can be used to determine the appropriate time required for the PMMA cements to restore its full functional capabilities for orthopedic or orthodontic surgeries.

The limitation of the study is that there was no study conducted to find the relation of curing time on exothermic behavior of cement, which could be important in understanding the residual stress build up due to the exothermic reaction. Such an experiment is important to develop a theoretical model to find a correlation between the residual stress due to curing and interface shear strength of implant/cement. The knowledge of understanding the curing effect on interface fracture strength of a titanium and cement is important for modeling the fracture behavior of titanium/cement interface due to thermal stress created by different curing temperatures.

## 5. Conclusions

The present investigation finds the following conclusion in relation to the research questions:

1. There exists a difference in internal morphology between HBC and VBC. In addition, there exist a difference of surface roughness of HBC and VBC along the interface of Ti/HBC and Ti/VBC. However, this study did not find any significant difference in the internal morphological characteristics due to the difference of curing time of HBC and VBC.
2. There exists a difference of curing time and exothermic temperature between HBC and VBC samples, which affected the flexural strength, hardness, and maximum exothermic temperature values between HBC and VBC samples.
3. There exists a difference of build-up residual load between Ti/HBC and Ti/VBC samples during the curing of each cement, which might affect the shear strength values under static and cyclic loading between Ti/HBC and Ti/VBC samples.

This study concludes that the mechanical characteristic of VBC is not comparable to HBC and needs improvement. Additionally, the cement curing time and temperature control the structural properties of cement and the bonding of the cement with the implant.

## Figures and Tables

**Table 1:** Flexural, hardness, and interface shear strength test results of HBC and VBC. The table shows the average, the standard of error, and the samples number used to find the values from tests.

Experimental Parameters	Sample Type	Curing Time	
		30 min	60 min
Flexural strength (MPa)	HBC	56.24 ± 2.55 (*n* = 6)	70.05 ± 1.90 (*n* = 6)
	VBC	61.60 ± 0.86 (*n* = 6)	65.90 ± 0.47 (*n* = 5)
Hardness (Rockwell R hardness number)	HBC	64 (*n* = 3)	79 (*n* = 3)
	VBC	42 (*n* = 3)	71 (*n* = 3)
Interface shear strength (MPa)	HBC	1.71 ± 0.09 (*n* = 5)	3.08 ± 0.07 (*n* = 4)
	VBC	0.85 ± 0.04 (*n* = 3)	1.08 ± 0.03 (*n* = 3)

**Table 2:** Surface roughness test results of HBC and VBC. The table shows average, standard of error, and samples number used to find the values from tests.

Experimental Parameters	Sample Type	Curing Time	
		30 min	60 min
Arithmetic mean height (µm)	HBC	0.16 ± 0.01 (*n* = 3)	0.18 ± 0.03 (*n* = 3)
	VBC	0.11 ± 0.00 (*n* = 3)	0.13 ± 0.01 (*n* = 3)
Root mean square height (µm)	HBC	0.23 ± 0.01 (*n* = 3)	0.24 ± 0.03 (*n* = 3)
	VBC	0.13 ± 0.01 (*n* = 3)	0.17 ± 0.01 (*n* = 3)
